# Measuring Coverage in MNCH: Accuracy of Measuring Diagnosis and Treatment of Childhood Malaria from Household Surveys in Zambia

**DOI:** 10.1371/journal.pmed.1001417

**Published:** 2013-05-07

**Authors:** Thomas P. Eisele, Kafula Silumbe, Josh Yukich, Busiku Hamainza, Joseph Keating, Adam Bennett, John M. Miller

**Affiliations:** 1 Department of Global Health Systems and Development, Tulane University School of Public Health and Tropical Medicine, New Orleans, Louisiana, United States of America; 2 Malaria Control and Evaluation Partnership in Africa, Program for Appropriate Technology in Health, Lusaka, Zambia; 3 National Malaria Control Centre, Lusaka, Zambia; Wellcome Trust Senior Research Fellow in Clinical Science, UCL Reader in International Child Health, Honorary Consultant, Great Ormond Street Hospital for Children, United Kingdom

## Abstract

**Background:**

To assess progress in the scale-up of rapid diagnostic tests and artemisinin-based combination therapies (ACTs) across Africa, malaria control programs have increasingly relied on standardized national household surveys to determine the proportion of children with a fever in the past 2 wk who received an effective antimalarial within 1–2 d of the onset of fever. Here, the validity of caregiver recall for measuring the primary coverage indicators for malaria diagnosis and treatment of children <5 y old is assessed.

**Methods and Findings:**

A cross-sectional study was conducted in five public clinics in Kaoma District, Western Provence, Zambia, to estimate the sensitivity, specificity, and accuracy of caregivers' recall of malaria testing, diagnosis, and treatment, compared to a gold standard of direct observation at the health clinics. Compared to the gold standard of clinic observation, for recall for children with fever in the past 2 wk, the sensitivity for recalling that a finger/heel stick was done was 61.9%, with a specificity of 90.0%. The sensitivity and specificity of caregivers' recalling a positive malaria test result were 62.4% and 90.7%, respectively. The sensitivity and specificity of recalling that the child was given a malaria diagnosis, irrespective of whether a laboratory test was actually done, were 76.8% and 75.9%, respectively. The sensitivity and specificity for recalling that an ACT was given were 81.0% and 91.5%, respectively.

**Conclusions:**

Based on these findings, results from household surveys should continue to be used for ascertaining the coverage of children with a fever in the past 2 wk that received an ACT. However, as recall of a malaria diagnosis remains suboptimal, its use in defining malaria treatment coverage is not recommended.

*Please see later in the article for the Editors' Summary*

## Editors' Summary

**Background:**

The World Health Organization estimates that there are over 200 million cases of malaria each year, with nearly 1 million deaths. The majority of these deaths are among children living in sub-Saharan Africa, and *Plasmodium falciparum* is the parasite responsible. Malaria transmission can be prevented by insect control measures, and current treatment regimens use antimalarial drugs. Recently, the use of highly effective artemisinin-based combination treatments (ACTs) has significantly reduced the deaths and disability caused by malaria. To avoid drug overuse and the development of parasite resistance to ACTs, the World Health Organization recommends that before treatment with ACTs, a laboratory test to confirm malaria should be performed. Rapid diagnostic tests (RDTs) allow health workers to diagnose malaria in settings lacking laboratory facilities, thus providing a method for improving malaria diagnosis and reducing the overuse of ACTs.

**Why Was This Study Done?:**

The success of RDTs and ACTs across Africa in combating malaria is measured by standardized national household surveys. These surveys assess the proportion of children with a fever in the past two weeks who have received an antimalarial treatment within 1–2 days of the onset of fever. The surveys do not distinguish between treatment of a suspected malaria case and one that was laboratory confirmed. Due to the availability and scale-up of RDTs in many African countries, caregivers and mothers are also now asked in national surveys if the child was tested for malaria, but are not usually asked for the result of any malaria diagnostic test given. Knowing whether a child has been diagnosed with malaria is necessary to construct a better indicator of what proportion of children receive an effective and appropriate antimalarial within the appropriate treatment time frame. This indicator is important because it provides more insight into the current diagnosis and treatment policies in most African countries.

Biased coverage estimates for diagnosis and treatment may result from these types of surveys because survey questions to caregivers of children concerning fever in the past two weeks, treatment-seeking behavior, and malaria diagnosis and treatment can be particularly subject to sources of error and bias. Despite this possibility, these indicators and surveys have not been checked against direct observation of the children to assess the validity of caregivers' recall and household surveys to gauge appropriate treatment of malaria in children.

**What Did the Researchers Do and Find?:**

In this study, the authors investigated the validity of caregiver recall of malaria diagnosis and treatment in children under five years old. The authors did a cross-sectional study of five public clinics in Kaoma District, Western Provence, Zambia, to estimate the sensitivity, specificity, and accuracy of caregivers' recall of malaria testing, malaria diagnosis, and antimalarial treatment, and compared the surveys to direct observation at the health clinics. The results from this study demonstrate low sensitivity of caregiver recall of malaria diagnostic use, test results, and malaria diagnosis among children who had a fever in the past two weeks. However, the accuracy of caregiver recall that a child received an ACT was relatively high in this setting. This suggests that the current indicator for measuring the coverage of children with a fever in the past two weeks who received an ACT can be applied in similar settings, and may be useful for estimating infection and treatment over time.

**What Do These Findings Mean?:**

These findings suggest that results from household surveys are accurate for obtaining information about the coverage of children with a fever in the past two weeks that receive an ACT. However, as caregiver recall of a malaria diagnosis is not highly sensitive, the authors suggest that malaria diagnosis from caregiver recall in household surveys is not recommended for defining malaria treatment coverage.

**Additional Information:**

Please access these websites via the online version of this summary at http://dx.doi.org/10.1371/journal.pmed.1001417.

- More information about malaria is available from MedlinePlus, the World Health Organization, and the US Centers for Disease Control and Prevention
- The Roll Back Malaria Partnership brings numerous organizations together to combat malaria around the globe

*This paper is part of the* PLOS Medicine *“Measuring Coverage in MNCH” Collection.*

## Introduction

New rapid diagnostic tests (RDTs) and artemisinin-based combination therapies (ACTs) are being scaled up across Africa in the fight against malaria [1],[2]. Nearly all endemic countries in Africa have now adopted a policy requiring laboratory confirmation of suspected malaria cases with either RDTs or microscopy [3]. Once cases are confirmed, ACTs are the current first-line drugs for treating uncomplicated *Plasmodium falciparum* malaria infection. These policies are critical to ensure that malaria cases are promptly and properly diagnosed and treated, especially as malaria transmission falls because of increasingly high coverage of effective vector control measures [4].

To assess progress in the scale-up of RDTs and ACTs across Africa, malaria control programs have increasingly relied on standardized national household surveys to assess the proportion of children with a fever in the past 2 wk who received an affective antimalarial within 1–2 d of the onset of fever [5]. This indicator makes no distinction between treatment of a suspected malaria case and one that was laboratory confirmed. Because of the availability and scale-up of RDTs in many African countries, caregivers and mothers are also now asked in national surveys if the child received a heel or finger stick (for an assumed test of malaria) and if they sought treatment at a health facility [6]. However, caregivers are not typically asked if they recall the result of any malaria diagnostic test given. Information on the test result would be needed to construct a better indicator of the proportion of children with a fever in the past 2 wk with a laboratory-confirmed malaria diagnosis who received an effective and appropriate antimalarial within 1–2 d of the onset of fever. This indicator would capture current diagnosis and treatment policies in most African countries better than the current standard.

Individuals' recall of autobiographical events, including past health events, is a complex process and can be biased by multiple external and internal factors, including the frequency of the event, time since the event, cues provided by interviewers or questionnaire design, and individuals' emotional perception of related events [7]. Survey questions to caregivers of children related to fever in the past 2 wk, treatment-seeking behavior, and malaria diagnosis and treatment are particularly subject to sources of error and bias, which may result in biased coverage estimates for diagnosis and treatment [8]. However, these indicators and their means of measurement to our knowledge have yet to be validated against a gold standard to assess the validity of caregivers' recall.

The objective of this study was to assess the validity of the primary coverage indicators for malaria diagnosis and treatment in children <5 y old, constructed from data collected during household surveys. To accomplish this, a cross-sectional study was conducted in five health clinics in Kaoma District, Western Provence, Zambia, to estimate the sensitivity, specificity, and accuracy of caregivers' recall of malaria testing, diagnosis, and antimalarial treatment compared to a gold standard of direct observation at the health clinics. The relationship between the accuracy of caregiver recall for malaria diagnosis and treatment and socio-demographic characteristics were also assessed.

## Methods

### Ethics Statement

Ethical approval for this study was obtained from the Institutional Review Boards of Tulane University, the University of Zambia, and the Program for Appropriate Technology in Health.

### Study Site

**Figure 1:**
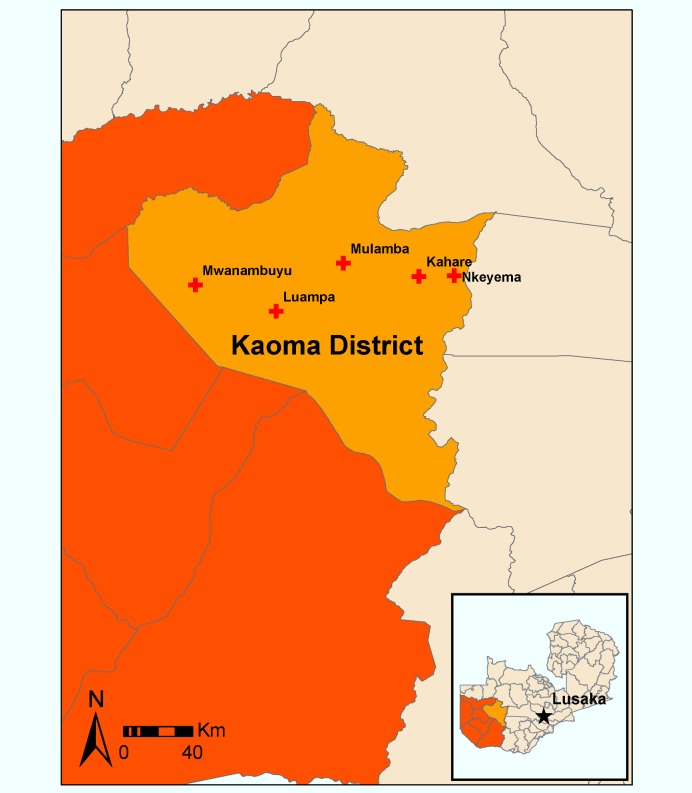
Map of study clinics in Kaoma District, Western Provence, Zambia.

The study was conducted in Kaoma District (population of approximately 183,000), located in Western Province about 460 km west of the capital city of Lusaka (Figure 1). Kaoma is a rural district, with an economy based primarily on commercial and subsistence farming. Kaoma, covering 23,000 km^2^, has a tropical climate, with seasonal rains lasting from November to April and a corresponding peak in malaria transmission from April to June.

This study took place in four rural public health facilities and one urban public health facility; all were purposefully selected for adequate patient flow of suspected malaria cases in children that received clinical and laboratory diagnoses. The estimated combined catchment area population of the five clinics was 42,100. Malaria burden in the district fluctuates widely during the course of the year with the transmission season. As a percentage of outpatient attendance, malaria diagnoses can range from 2% during the dry season to more than 60% during the transmission season. Malaria test positivity rates among clinic attendees similarly can fluctuate from less than 5% during the dry season to as high as 60%–70% during the peak of the transmission season in April–June. As per national policy in Zambia, artemether/lumefantrine (ACT) was the first-line drug combination used in all five clinics for treating uncomplicated malaria [3]. Although the national policy in Zambia calls for 100% laboratory diagnosis of suspected malaria cases, the clinic in Mulamba primarily relied on clinical diagnosis (diagnosis based on symptoms) because of a stock-out of RDTs throughout the study period; Mulamba does not have microscopy. The remaining four clinics almost exclusively used laboratory diagnosis with RDTs.

### Study Design, Participants, and Data Collection Procedures

A cross-sectional study design was used to assess the validity of the primary coverage indicators for malaria diagnosis and treatment from data collected using household surveys. Data collection at the clinics and at home-based follow-ups occurred in May–June 2012. All caregivers at least 18 y old of children <5 y old presenting for treatment for fever in the five health centers during the study period were eligible for inclusion in this study.

Details of the clinic visit for the episode, including the child's age, presence of fever, diagnostic used (if any), test result, diagnoses made, and treatment provided, were recorded in specially designed clinic visit sheets. These forms were completed for each child at the conclusion of the visit by the attending health professional. While the health professionals received training in completing the forms by the study team prior to the start of the study, they were not informed explicitly that this study aimed to assess caregiver recall of the diagnoses and treatments received at the child's visit. The clinic visit sheets captured all relevant demographic, diagnosis, and treatment information that is typically captured in the standard clinic logbook.

At the conclusion of the clinic visit, caregivers were asked if they would like to participate in a malaria study in their community by the attending health professional. If they agreed, a study representative at the clinic obtained their contact information so that a follow-up visit at their house could be scheduled. To replicate national surveys that ask a series of questions to mothers (Demographic and Health Surveys [DHS] and Malaria Indicator Surveys [MIS]) and caregivers (Multiple Indicator Cluster Surveys [MICS]) of children <5 y old with a fever in the past 2 wk related to malaria diagnosis and treatment, follow-up home visits were scheduled 0–14 d following the clinic visit. Follow-up visit dates were randomly assigned 0–14 d using a random number generator at the time of participant recruitment, and a follow-up appointment time and date were arranged with the caregiver for the follow-up interview. Clinic visit sheets were stapled to the contact information sheet for each caregiver that agreed to a follow-up interview.

Informed consent was obtained from all caregivers at the start of the home-based follow-up interviews. For the majority of children, caregivers were identified as the mother of the child identified at the clinic visit, and as listed on the contact information sheet. If the mother of the child who sought treatment at the clinic did not live in the house, the caregiver of the child was identified and interviewed. If the caregiver did not consent to be in the study at the time of the interview, the contact sheet and clinic visit sheet were destroyed and participation in the study was discontinued. For consenting caregivers, a standardized questionnaire based on the DHS survey [9] and MIS survey (and similar to the MICS survey) was used to obtain information on whether the child had a fever in the past 2 wk; whether treatment was sought, including when and where; whether a diagnostic test was given, based on recall of a finger or heel stick; and details of the treatment given. Questions about recall of the results of a malaria test (if a finger/heel stick was recalled) and about whether any malaria diagnosis was made were added to the questionnaire. No props or cues were used in asking caregivers about the diagnosis and treatment the child may have received. Information on household assets, as well as the caregiver's sex, age, education level, and literacy, was also obtained based on the questions used in the DHS and MIS surveys. Data were entered into specially designed personal digital assistants used for the Zambia national MIS survey.

### Primary Outcome and Explanatory Variables

The primary outcomes were the sensitivity, specificity, and accuracy of caregivers' recall of the child having received a finger/heel stick (for malaria laboratory diagnostic), result of the malaria diagnostic test, diagnosis for the fever episode, and type of treatment received. Recall of events related to the treatment of the child's fever was ascertained from the follow-up questionnaire. Data recoded on the clinic visit sheet served as the gold standard against which caregivers' recall was assessed. Sensitivity represents the percent of caregivers that correctly recalled the child's receiving a finger/heel stick, a test result, a malaria diagnosis, and a particular antimalarial treatment, of those that actually received them based on clinic observation. Specificity represents the percent of caregivers that correctly recalled the child's not receiving a finger/heel stick, a test result, a malaria diagnosis, and a particular antimalarial treatment, among those that truly did not receive them based on clinic observation. Accuracy represents the percent of caregivers whose recall of the diagnosis and treatment received for their child's fever episode agreed with the clinic observation.

Household socioeconomic status was derived from a principal components analysis of household assets using previously established methods, divided into wealth quintiles [10]. Caregiver education was categorized as none, at least some primary, or secondary or higher. Caregiver age was categorized as 18–24, 25–34, 35–44, or 45 y or older. All caregiver demographic and social data were obtained from the follow-up questionnaire. Days to follow-up was the difference between the clinic attendance date and the home follow-up interview date, disaggregated by 0–6 and 7–14 d.

### Sample Size and Stratification

A total sample size of 600 caregivers of children presenting with fever at the five study clinics was sought, stratified equally by those with a laboratory-confirmed malaria diagnosis, those with a laboratory-negative malaria diagnosis, and those without a laboratory diagnosis. The sample size for estimating the sensitivity and specificity of caregiver recall of diagnostic result, malaria diagnosis, and antimalarial treatment was based on the probability of committing a type 1 error of 5% (two-tailed test), a sensitivity of 70%–80%, a specificity of 80%–90%, a precision of ±7.0%, and an interview refusal rate of 10%, resulting in a sample size of 200 caregivers of children who tested positive for malaria and 200 caregivers of children who tested negative for malaria. Children across the five study clinics presenting with fever were selected accordingly, based on stratification by malaria diagnosis. To assess the sensitivity and specificity of caregiver recall of whether the child received a finger/heel stick for malaria diagnosis, an additional 200 caregivers of children who did not receive a finger/heel stick were selected, based on the same parameters outlined above.

### Statistical Analysis

For the primary outcomes related to measuring the coverage of malaria diagnosis and treatment, SAS 9 was used to obtain estimates of sensitivity, specificity, and accuracy, along with 95% confidence intervals (CIs). Standard errors and accompanying 95% CIs for all descriptive point estimates and outcomes were estimated with the Huber–White Sandwich estimator to account for correlated data at the health facility level.

Differences in sensitivity, specificity, and accuracy of malaria diagnosis and treatment between clinics, child characteristics, and caregiver socio-demographic characteristics were tested using chi-square (χ^2^). Fisher's exact test was used where any cell count was below ten. Separate logistic regression models were used to assess the association of child and caregiver characteristics with the outcomes of sensitivity, specificity, and accuracy for malaria diagnosis and treatment. As there was considerable heterogeneity across the five clinics with respect to malaria diagnosis, logistic regression models were constructed using both clinic as a random effect and clinic as an independent cluster in generalized estimating equation models. The models with clinic as a random effect yielded more conservative estimates than the generalized estimating equation models in all instances, and were thus chosen to account for heterogeneity across the five health facilities. The final models included child age in years, sex, days to follow-up, caregiver's age category, caregiver's relationship with child (mother or not), caregiver's education, and household wealth quintile, with clinic included as a random effect. Diagnostic test (laboratory versus clinical) was included in models for the outcomes of recall of a positive malaria diagnosis and whether the child was given ACT.

Using the observed sensitivity and specificity in this study for caregiver recall of finger/heel stick, positive diagnostic result, positive malaria diagnosis (laboratory confirmed or clinical), and ACT given, estimates for the coverage of these interventions that one would expect from a household survey of caregivers' recall were modeled across true intervention coverages (observed at clinic) ranging from 0% to 100% as follows: estimated coverage from caregiver recall = (true coverage at clinic*×*sensitivity)+[(1−true coverage at clinic)*×*[1−specificity)]. Thus, at zero intervention coverage at a clinic (e.g., no finger/heel stick available/offered), the estimated coverage from caregiver recall is equal to the observed 1 – specificity, while at 100% coverage at the clinic, the estimated coverage from caregiver recall is equal to the observed sensitivity. Random sensitivity and specificity (i.e., 50% each) would be expected to yield 50% intervention coverage from caregiver recall, irrespective of the true coverage at a clinic.

## Results

A total of 644 caregivers of children seeking treatment at the five outpatient study clinics were asked to participate in the study. A total of 601 caregivers agreed to be followed up, consented to take part in the study at the time of the follow-up interview, and completed the questionnaire, resulting in a nonresponse rate of 6.7%.

Nearly all (96.5%) of the 115 children diagnosed with malaria in the Mulamba clinic received a diagnosis based on clinical examination rather than on the results of a laboratory test (Table 1). Nearly all (98.3%) of malaria diagnoses in children in the other four study clinics were based on an RDT (one based on microscopy). Among children <5 y old seeking treatment across the five study clinics, if they received a malaria diagnosis, it was based on a laboratory test 67.4% of the time. Of children with a malaria diagnosis, nearly all (97.7%) received an antimalarial, of which 92.1% received the ACT Coartem.

**Table 1:** Characteristics of the children, caregivers, and clinics included in the study, Western Province, Zambia, 2012.

Characteristic	Number	Point Estimate (Percent)	95% CI	Sample Size
**Child age in years** a				601
0	145	24.1	20.8–27.4	
1	168	28.0	17.9–38.0	
2	121	20.1	17.4–22.8	
3	94	15.6	11.6–19.6	
4	73	12.1	6.5–17.8	
**Female child** a	304	50.6	45.8–55.4	601
**Age of caregiver in years** a				601
18–24	247	41.1	31.6–50.6	
25–34	241	40.1	34.6–45.6	
35–44	97	16.1	13.0–19.3	
45 or older	16	2.7	0.0–6.7	
**Female caregiver** a	573	95.3	89.2–100.0	601
**Mother of child as caregiver** a	561	93.3	82.5–100.0	601
**Caregiver interviewed same as who took child to clinic visit** a	575	95.7	88.9–100.0	601
**Caregiver education** a				601
None	42	7.0	1.1–12.9	
At least some primary	346	57.6	39.2–75.9	
Secondary or higher	213	35.4	15.2–55.7	
**Mean number of days between clinic visit and survey interview** a		5.2	2.8–7.5	601
**Proportion of observations from each clinic**				601
Kahare	114	19.0	15.8–22.1	
Luampa	101	16.8	13.8–19.8	
Mulamba	149	24.8	21.3–24.1	
Mwanambuyu	125	20.8	17.5–24.1	
Nkeyema	112	18.6	15.5–21.8	
**Proportion of malaria diagnoses that were laboratory confirmed**				
Kahare	48	100	—	48
Luampa	42	97.7	93.0–100	43
Mulamba	4	3.5	0.1–6.9	115
Mwanambuyu	61	95.3	90.0–100	64
Nkeyema	83	100	—	83

a Standard errors estimated using the Huber–White Sandwich estimator to account for correlated data at the facility level.

The vast majority (93.3%) of caregivers interviewed at follow-up were the mother of the child, with nearly all (95.7%) being the same person who took the child to the clinic visit (Table 1). Nearly all (95.3%) caregivers interviewed at their homes for the follow-up visit were female, the majority were less than 34 y old, and most had at least some primary school, with a third having attended secondary school or higher (Table 1).

Differences in socio-demographic characteristics of children and caregivers across the five clinics are shown in Table S1. Children were similar across the five study clinics with respect to age and sex (Table S1). Caregivers were similar across study clinics in age, but varied slightly across clinics by sex, relationship to child, education, and household wealth.

Nearly all (96.0%) of the 601 children included in the study were reported to have had a fever in the past 2 wk by the caregiver at the follow-up interview (Table 2). Based on clinic observation, two-thirds (66.9%) of all children were given either an RDT or microscopy (only one child received microscopy) to test for malaria. Of those tested (*n = *402), over half (58%) had a positive test result. Of all children (*n = *601), over half had a diagnosis of malaria at the clinic visit, either laboratory confirmed or clinically diagnosed.

**Table 2:** Characteristics of treatment recall by caregivers and observation at study clinics, Western Province, Zambia, 2012.

Characteristic	Number	Point Estimate (Percent)	95% CI	Sample Size
**Caregiver recall for household interview**				
Child with fever in past 2 wk	577	96.0	86.8–100.0	601
Child taken for treatment for fever, of those with fever in past 2 wk	390	67.6	33.8–100.0	577
Child received finger/heel stick	263	43.7	0.0–89.5	601
Child tested for malaria with RDT	259	43.1	0.0–89.0	601
Child tested for malaria with microscopy^a^	1	0.2	0–0.6	601
Of those for whom caregiver recalled child being tested, caregiver had result shared with them	234	90.0	79.6–100.0	260
Of those for whom caregiver recalled child being tested, child with positive test	175	67.3	39.9–94.7	260
Child with malaria diagnosis^b^	321	53.4	40.3–66.5	601
Child given any antimalarial	328	54.6	19.6–89.5	601
Child given ACT	304	50.6	12.2–89.0	601
**Observed at clinic**				
Child tested for malaria with RDT	401	66.7	12.2–100.0	601
Child tested for malaria with microscopy^a^	1	0.2	0–0.6	601
Of tested, child with positive test	233	58.0	40.2–75.7	402
Child with malaria diagnosis^b^	353	58.7	37.0–80.4	601
Child given any antimalarial	381	63.4	43.2–83.6	601
Child given ACT	351	58.4	34.2–82.6	601

All standard errors estimated using the Huber–White Sandwich estimator to account for correlated data at the facility level.

a Only one child tested with microscopy.

b Includes laboratory-confirmed malaria and clinical diagnosis based on symptoms.

During the home interviews, only two-thirds (67.6%) of caregivers recalled that the child was taken to a health facility for a fever in the past 2 wk (among those children with a fever in the past 2 wk, *n = *577; Table 2). This recall did not vary by whether the caregiver was the mother of the child or someone else in the household (*χ^2^* = 0.70, df = 1, *p* = 0.4033). However, the caregiver was significantly more likely to have correctly recalled that the child was taken for treatment for the fever if they were the same person who took the child to the clinic (*χ^2^* = 5.0, df = 1, *p* = 0.0279).

The sensitivity, specificity, and accuracy of recalling whether the child received a finger/heel stick, a positive laboratory test result, a positive malaria diagnosis, and treatment with an ACT varied significantly across the five study clinics (Tables S2 and S3). While no clear pattern emerged, caregivers of children seen at the Mwanambuyu clinic had the most accurate recall of these events, while caregivers from the Kahare, Luampa, and Mulamba clinics had the least accurate recall of these events.

Of all children sampled, caregivers' recall of their child receiving a finger or heel stick was nearly identical to their recall that the child was tested for malaria with a laboratory test (43.7% and 43.3%, respectively; Table 2). Of children tested (*n = *260), 90.0% of their caregivers reported that the results were shared with them, with two-thirds (67.3%) reporting that the test was positive. Among all children, over half the caregivers (53.4%) recalled that a malaria diagnosis (laboratory confirmed or clinical) was made, and over half (50.6%) reported that the child received an ACT.

Of children with fever in the past 2 wk (*n = *577), the sensitivity for recalling that a finger/heel stick was done was 61.9% (95% CI 58.1%–67.7%), with a specificity of 90.0% (95% CI 85.7%–94.2%); results of recalling whether a malaria laboratory test was performed were nearly identical. Based on the random effects logistic regression (Table S4), there was no association between caregiver recall of a finger/heel stick and child and caregiver characteristics.

Compared to the gold standard of clinic observation, for children with fever in the past 2 wk who received a laboratory diagnosis at the clinic, the sensitivity for recalling that it was positive was 62.4% (95% CI 56.1%–68.7%), with a specificity of 90.7% (95% CI 86.3%–95.2%). Table S4 presents the random effects logistic regression models assessing factors related to the sensitivity, specificity, and accuracy of a caregivers' recall. The accuracy of recall of a positive test result was also significantly higher in caregivers with secondary or higher education, as compared to those with just primary education.

Among all children with a fever in the past 2 wk, the sensitivity of recalling that the child was diagnosed with malaria, irrespective of whether a laboratory test was actually done, was 76.8% (95% CI 72.4%–81.3%), with a specificity of 75.9% (95% CI 70.4%–81.4%). Based on logistic regression (Table S4), the sensitivity of recalling that a malaria diagnosis was made was significantly better when a laboratory diagnosis was used, as compared to clinical diagnosis (adjusted odds ratio = 1.4, 95% CI 1.3–1.6).

Compared to clinic observation, among children with a fever in the past 2 wk, the sensitivity and specificity for recalling that an ACT was given were 81.0% (95% CI 76.8%–85.2%) and 91.5% (95% CI 87.9%–95.1%), respectively, resulting in an accuracy of 85.3% (95% CI 82.4%–88.2%; Table 3). Recall that any antimalarial was given was nearly identical. Logistic regression showed that the accuracy of recalling that an ACT was given was significantly better when the child received a laboratory diagnostic test (adjusted odds ratio = 1.2, 95% CI 1.1–1.3), as compared to clinical diagnosis (Table S4).

**Table 3:** Accuracy of caregiver recall of key questions of diagnosis and treatment of malaria, Western Province, Zambia, 2012.

Caregiver Recall	TP	TP+FN	Sensitivity (Percent)	95% CI	TN	TN+FP	Specificity (Percent)	95% CI	TP+TN	TP+TN+FP+FN	Accuracy (Percent)	(95% CI)
Recall of fever in past 2 wk	577	601	96.0	(86.8–100.0)	0	0	100.0	—	577	601	96.0	(86.8–100.0)
Recall of finger/heel stick^a^	244	388	62.9	(17.8–100.0)	170	189	90.0	(85.8–94.1)	414	577	71.8	(41.0–100.0)
Recall of malaria laboratory diagnosis performed^a^	240	388	61.9	(16.6–100.0)	170	189	89.4	(84.2–95.7)	410	577	70.9	(39.9–100.0)
Recall of positive malaria test result (of those tested at clinic)^a^	141	226	62.4	(14.2–100.0)	147	162	90.7	(75.6–100.0)	288	388	74.2	(49.6–98.9)
Recall that malaria diagnosis was made^b^	265	345	76.8	(55.1–98.5)	176	232	75.9	(48.3–100.0)	441	577	76.4	(55.2–97.6)
Recall of any antimalarial given^a^	305	372	82.0	(53.2–100.0)	182	205	88.8	(76.5–100.0)	487	577	84.4	(69.5–99.3)
Recall of ACT given^a^	277	342	81.0	(50.8–100.0)	215	235	91.5	(80.3–100.0)	492	577	85.3	(72.7–97.8)

All standard errors estimated using the Huber–White Sandwich estimator to account for correlated data at the facility level.

a Among children reported by caregiver to have a fever in the past 2 wk.

b Includes laboratory-confirmed malaria and clinical diagnosis based on symptoms.

CI, confidence interval; FN, false negative; FP, false positive; TN, true negative; TP, true positive.

**Figure 2:**
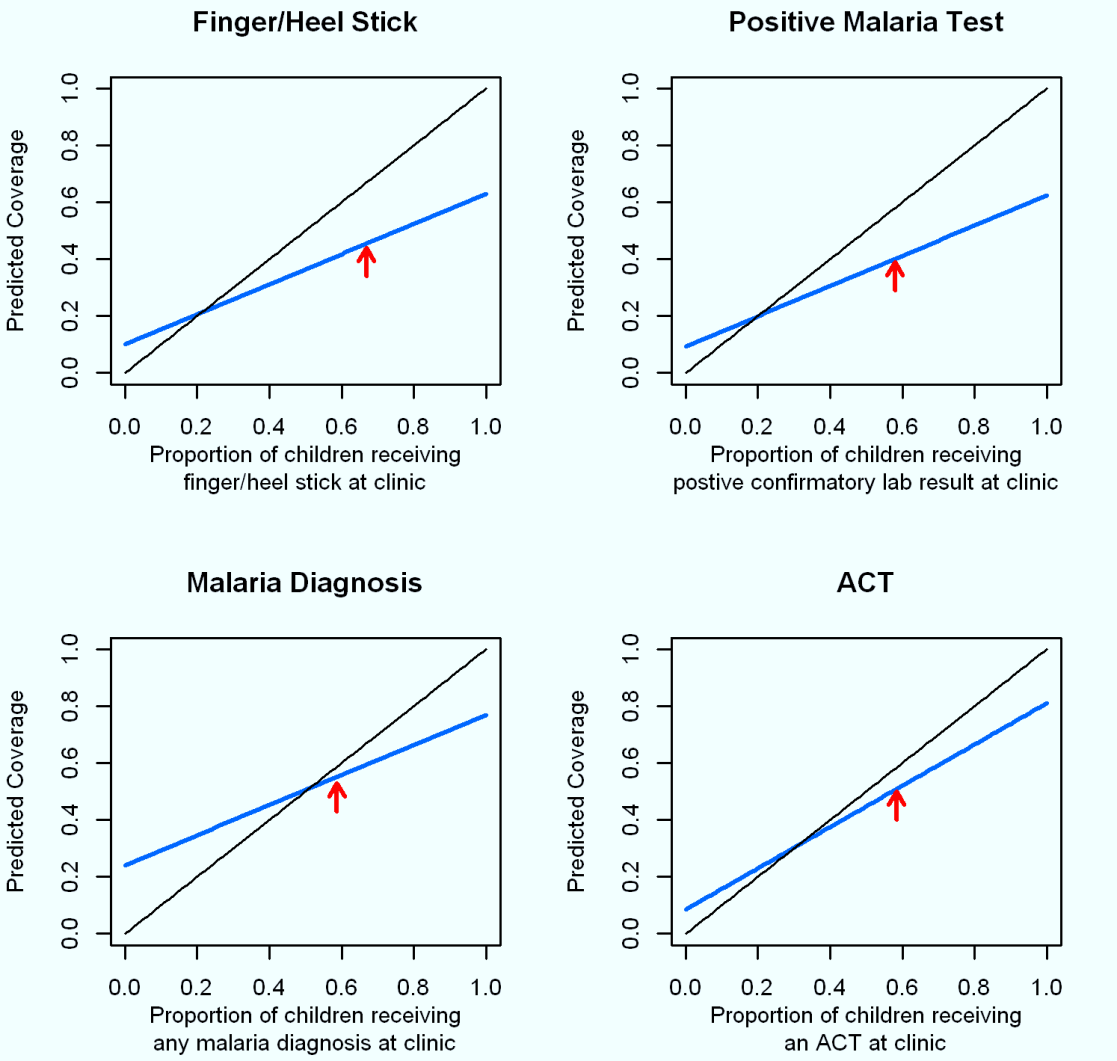
Modeled diagnosis and treatment coverage based on sensitivity and specificity of caregiver recall across actual intervention coverages in a given community. Proportions of patients actually experiencing each event at the study clinics are illustrated with red arrows. The solid black line at a 45° angle represents 100% sensitivity and specificity. Estimates for the coverage of these interventions expected from a household survey from caregiver recall with the sensitivity and specificity observed in this study (blue line) were modeled for true intervention coverages (observed at clinic) ranging from 0% to 100% as follows: estimated coverage from caregiver recall = (true coverage at clinic*×*sensitivity)+[(1−true coverage at clinic)*×*[1−specificity)].

The modeled coverage of finger/heel stick ascertained from caregiver recall at various coverage scenarios, based on the sensitivity and specificity observed in this study, shows that at low coverage levels, caregiver recall would tend to only slightly overestimate the true coverage of finger/heel sticks at clinics (Figure 2). However, where about 80% of children with a fever actually receive a finger/heel stick at clinics, the low sensitivity of caregiver recall of this happening would lead to a substantial underestimation of the coverage of this indicator. Modeled estimates of the recall of a positive malaria diagnosis based on the accuracy of caregivers' recall show this indicator to perform poorly, with substantial overestimation when the true level of malaria diagnosis in clinics is low, and substantial underestimation when positive malaria diagnoses are common (i.e., above 50%). The modeled estimates of the recall that a child with a fever received an ACT based on the accuracy of caregivers' recall performed much better, with only a slight overestimation when actual prescription of ACT is low (i.e., below 20%), while yielding a slight underestimation when prescription of ACT is more common.

## Discussion

A cross-sectional study was conducted in five rural health clinics in Kaoma District, Zambia, to estimate the sensitivity, specificity, and accuracy of caregivers' recall of malaria testing, malaria diagnosis, and antimalarial treatment. Observation of malaria diagnosis and treatment at the clinics served as the gold standard.

Overall, the sensitivity of caregiver recall that a malaria diagnostic test was performed on a child reported to have had a fever in the past 2 wk was poor in this setting, irrespective of whether the questionnaire asked if a finger or heel stick was done (62.9%) or whether a blood test for malaria was done (61.9%). Estimates of specificity for these questions were higher, at 90.0% and 89.4%, respectively. These results mean that about four out of ten respondents do not recall that a malaria diagnostic test was done when in fact the child received one, while relatively few (one in ten) incorrectly recall that a diagnostic was done when in reality one was not done. These results suggest that while there is substantial recall error (i.e., forgetting a child received a test), there is limited information bias related to stating a child received something he or she did not really receive. These results further suggest that the indicator used for measuring the coverage of laboratory diagnosis of malaria among children with a fever in the past 2 wk will yield substantial underestimates of RDT and microscopy coverage, within similar settings. This finding is consistent with the 2010 Zambia MIS, which showed a lower estimate of children receiving a finger or heel stick than what would be expected based on the programmatic data on the availability and use of RDTs across public facilities in Zambia [6].

Of those children that actually received a laboratory diagnosis, the sensitivity (62.4%) and specificity (90.7%) of recalling a positive test result were very similar to those of recalling that the test was done. This suggests that many caregivers fail to recall a positive test result, while relatively few state the child had a positive test result when they actually did not.

Among children with a fever in the past 2 wk, irrespective of whether a laboratory or clinical diagnosis was used at the clinic, there was relatively poor sensitivity (76.8%) and specificity (75.9%) of caregiver recall that a positive malaria diagnosis was made. The sensitivity and specificity of caregiver recall of a positive malaria diagnosis were significantly lower in the Mulamba clinic, which primarily based malaria diagnosis on clinical symptoms because of a stock-out of RDTs. This coincides with the finding that a laboratory diagnosis results in significantly better sensitivity (86.6%) of caregiver recall of a positive malaria diagnosis, as compared to a clinical diagnosis (57.0%). While the sensitivity of caregiver recall of a malaria diagnosis may improve as RDTs are scaled up across Africa and more children receive laboratory diagnoses, these results clearly suggest that more can be done to ensure physicians and nurses share the child's diagnosis with the caregiver at the visit.

Within this setting, the sensitivity (81.0%) and specificity (91.5%) of caregiver recall that the child received an ACT was reasonably high, resulting in an overall accuracy of 85.3%. These results suggest that the indicator for measuring the coverage of children with a fever in the past 2 wk who received an ACT may yield reasonable estimates. However, this study included only public clinics in Zambia where ACTs are supplied free of charge and where a concerted effort has been made to limit the use of monotherapies, such as sulfadoxine-pyrimethamine and chloroquine, for treating uncomplicated malaria in children [3],[11]. The estimates of sensitivity and specificity of ACT recall observed in this setting may be considerably higher than in other settings where ACTs are not free and where substantial use of antimalarial monotherapies persists; use of antimalarial monotherapies may be especially high in settings where a large share of children are taken to private facilities [12].

The Mwanambuyu clinic had consistently higher accuracy of recall of a finger/heel stick, a positive malaria test result, malaria diagnosis, and whether the child received an ACT (Tables S2 and S3). We surmise this was likely due to the population of the clinic having a higher education level (Table S1).

The standard recall period for asking about details of treatment of fevers in children in the DHS and MICS surveys is 2 wk. In this study setting, there was no observed drop-off in the sensitivity, specificity, and accuracy of caregiver recall of malaria diagnosis and treatment at a clinic visit that occurred 0–6 d prior to the survey interview compared to one that occurred 7–14 d prior. This suggests that there would be no benefit of improving the accuracy of caregiver recall by limiting survey questions to children that had a fever within a shorter time frame than the previous 2 wk.

The DHS survey asks details about a child's fever episode from the mother, while the MICS survey asks these details from the mother, if available, or, if the mother has died or does not live at the house, from a caregiver who is present in the house [13]. It is helpful that results from this study show little if any difference between the accuracy of recall of malaria diagnosis and treatment based on whether the caregiver is the mother or someone else in the house that cares for the child. This reinforces the comparability between the DHS and MICS surveys for measuring malaria treatment coverage indicators.

Several similar studies published as companion papers in this Collection measured the validity of indicators from household coverage surveys in estimating the prevalence of health events [14],[15]. Coinciding with our results of poor recall of malaria diagnosis, results from the study of childhood pneumonia prevalence and antibiotic treatment prevalence generally showed poor sensitivity and specificity for the classification of “true pneumonia” as opposed to “suspected pneumonia” or “symptoms of acute respiratory infection” and, subsequently, appropriate antibiotic treatment [14] While there is some evidence that caregiver recall of past health events may be highly sensitive and specific in some instances, including mothers' recall of child vaccination status [16], several studies have shown recall of previous health events to be quite poor and often biased. A study of recall of essential drug treatment dosage and duration in rural Burkina Faso showed that while approximately 70% of patients interviewed after initial consultation could remember the dosage of drugs, only 30% recalled the duration of appropriate treatment [17]. While our study showed that caregivers recalled a child receiving an ACT with relatively high sensitivity and specificity, the results on other indicators, including whether or not the child received a malaria diagnostic test and the results of the test, were much less promising. This indicates that it will be difficult to estimate accurately the proportion of children receiving appropriate malaria treatment with household survey methods. Though our study found high sensitivity and specificity of fever recall, this may be due to interviewer bias or to participation in the study positively influencing recall. Measures of fever, clinic attendance, and antimalarial prescription recall conducted in Demographic Surveillance System settings in western Kenya indicated that fever recall over a 2-wk period underestimates the true prevalence because of recall bias, and that the bias tends to increase when the time from the symptom to the interview increases; such changes were more pronounced among caregivers than among adult patients [18]. That study also showed similar results for documented clinic attendances, antimalarial prescriptions, and antibiotic prescriptions. Furthermore, differences in questionnaire design may also influence the results for recall of past health events; a study of antimalarial drug utilization recall conducted in Mozambique indicated that recall of which antimalarial drug was used to treat a past episode of malaria could be influenced by the ordering of the drugs as read to the respondent [19].

Results from this study should be treated with caution for several reasons. First, this study may have limited broad external validity across Zambia and other African settings for several reasons, and results may actually represent a best-case scenario for the accuracy of caregiver recall of malaria diagnosis and treatment events. This study was conducted among only five public health clinics in a single rural district of Zambia selected by convenience; they are not representative of all of Zambia. Zambia has achieved substantial scale-up of malaria control measures with considerable resources from the government and international donors [20]. One hundred percent laboratory confirmation of suspected malaria is encouraged under the current national diagnosis and treatment policy, with substantial resources allocated to scaling up use of RDTs since 2007 [3]. Compared to many other African settings, there may have been fewer stock-outs of ACTs. Second, while details of the study aims and objectives were not explicitly presented to the clinic staff, it is possible their behaviors were influenced by a “malaria study” being conducted, which may have influenced caregiver recall. Similarly, while caregivers recruited into the study were not informed of the exact details of the study until the informed consent was obtained at the start of the follow-up interview, it is possible that by agreeing to a follow-up at their home for a “malaria study,” their recall of events at the clinic may have been biased. Third, while the information in the clinic visit sheets was entered by the attending health professionals themselves, we were not able to validate this information against a secondary direct observation. And last, props, such as the box that Coartem (artemether/lumefantrine) most commonly comes in or commonly used RDT cassettes, were not used during the survey interviews. It is possible the lack of such props, which are commonly used in many national surveys, may have hindered the accuracy of caregiver recall [7].

### Conclusions and Recommendations

While specificity of caregiver recall was reasonable, compared to a gold standard of direct observation at clinics, results from this study show there to be relatively poor sensitivity of caregiver recall of receiving a malaria diagnostic test and having the diagnostic test result shared, for children with a fever in the past 2 wk. These results suggest that at the current low coverage levels of malaria diagnosis across most African countries, estimates of diagnostic coverage may be reasonable (with slight overestimation due to less than perfect specificity), but as coverage in the population increases, survey estimates will yield increasingly larger underestimates of the true coverage because of the poor sensitivity of caregiver recall. Using household surveys to measure trends in the population coverage of laboratory diagnostics would therefore mask the true program success where high coverage of access to laboratory diagnoses is achieved. Recall of malaria diagnosis for children with a fever, irrespective of whether a laboratory diagnosis was made, had poor sensitivity and specificity, rendering this a very poor indicator for program managers to assess trends over time for this indicator. However, the accuracy of caregiver recall that a child received an ACT was relatively high in this setting, suggesting that the current indicator for measuring the coverage of children with a fever in the past 2 wk who received an ACT may yield reasonable estimates in similar settings, and may prove useful for measuring trends over time.

Based on these findings, results from surveys should continue to be used for ascertaining the coverage of children with a fever in the past 2 wk that received an ACT. However, as recall of a malaria diagnosis remains suboptimal, its use in defining malaria treatment coverage is not recommended.

As laboratory diagnosis is scaled up, these results suggest recall of any malaria diagnosis may improve; continued research to assess changes in malaria diagnosis recall as RDTs are scaled up is recommended. In the meantime, better communication between health professionals and caregivers should be promoted through additional training, with a focus on communicating diagnostics used, the child's diagnosis, and the treatment provided. For tracking progress towards targets for prompt, effective treatment of malaria, it is recommended that program managers and policy makers use household survey data only for measuring coverage of treatment seeking for fevers and access to antimalarial drugs. If possible, these data should then be supplemented with data from health system programs on the proportion of suspected malaria cases that receive a laboratory malaria diagnostic test, and the proportion of suspected and laboratory-confirmed malaria cases that receive the appropriate antimalarial. Where possible, studies consisting of exit interviews with caregivers following fever consultations would also prove useful for estimating the proportion of children receiving appropriate malaria diagnosis and treatment at the health system level.

## Supporting Information

Table S1 **Characteristics of the children, caregivers, and households, by clinic, Western Province, Zambia, 2012.**
(DOC)

Table S2 **Accuracy of caregiver recall of key questions of diagnosis and treatment of malaria for children with reported fever in the past 2 wk, by follow-up and social-demographic characteristics, Western Province, Zambia, 2012.**
(DOC)

Table S3 **Bivariate statistical test for differences of accuracy of caregiver recall of key questions of diagnosis and treatment of malaria for children with reported fever in the past 2 wk, by follow-up and social-demographic characteristics, Western Province, Zambia, 2012.**
(DOC)

Table S4 **Random effects logistic regression models of sensitivity, specificity, and accuracy of caregiver recall of key questions of diagnosis and treatment of malaria for children with reported fever in the past 2 wk: associations with follow-up and social-demographic characteristics, Western Province, Zambia, 2012.**
(DOC)

## Abbreviations

ACT: artemisinin-based combination therapy
CI: confidence interval
DHS: Demographic and Health Surveys
MICS: Multiple Indicator Cluster Surveys
MIS: Malaria Indicator Surveys
RDT: rapid diagnostic test
