# Supplementary material for: Measuring Coverage in MNCH: Accuracy of Measuring Diagnosis and Treatment of Childhood Malaria from Household Surveys in Zambia
Source: PLoS Med. 2013 May 7;10(5):e1001417. doi: 10.1371/journal.pmed.1001417 (PMC3646207; doi:10.1371/journal.pmed.1001417)
Supplement: Table S1 — Characteristics of the children, caregivers, and households, by clinic, Western Province, Zambia, 2012. (DOC) [file pmed.1001417.s001.doc]

**Table S1: Characteristics of the children, caregivers and households, by clinics, Western Province Zambia 2012**

| n = 601 |  | | **Point estimates** | |  | |  | |
| --- | --- | --- | --- | --- | --- | --- | --- | --- |
| **Participant characteristic** | Kahare | Luampa | | Mulamba | Mwanambuyu | Nkeyema | | Chi-square test statistic, df  (p-value) |
| Child age in years |  |  | |  |  |  | |  |
| 0 | 24.6 | 21.8 | | 25.5 | 27.2 | 15.9 | |  |
| 1 | 19.1 | 39.6 | | 20.1 | 33.6 | 21.4 | |  |
| 2 | 19.8 | 17.8 | | 22.2 | 17.6 | 21.4 | |  |
| 3 | 14.2 | 11.9 | | 15.4 | 15.2 | 21.4 | |  |
| 4 | 12.3 | 8.9 | | 16.8 | 6.4 | 15.2 | | 25.2, 16 (0.0678) |
| Percent children female | 56.1 | 52.5 | | 50.3 | 45.6 | 49.1 | | 2.90, 4 (0.5754) |
| Age of caregivers in years |  |  | |  |  |  | |  |
| 18-24 | 43.9 | 50.5 | | 32.2 | 36.8 | 46.4 | |  |
| 25-34 | 36.0 | 35.6 | | 45.0 | 43.2 | 38.4 | |  |
| 35-44 | 19.3 | 12.9 | | 15.4 | 18.4 | 14.3 | |  |
| 45-54 | 1.0 | 1.0 | | 7.4 | 1.6 | 1.0 | | 12.4, 8 (0.1339) |
| Percent caregivers female | 89.5 | 99.0 | | 91.3 | 100 | 98.2 | | 25.6, 4 (<0.001) |
| Percent caregiver mother of child | 88.6 | 100 | | 82.6 | 100 | 99.1 | | 54.2, 4 (<0.001) |
| Caregiver education (%) |  |  | |  |  |  | |  |
| None | 3.5 | 10.9 | | 10.7 | 8.8 | 0 | |  |
| At least some primary | 57.9 | 53.5 | | 48.3 | 81.6 | 46.4 | |  |
| Secondary or higher | 38.6 | 35.6 | | 40.9 | 9.6 | 53.6 | | 68.6, 8 (<0.001) |
| Household wealth |  |  | |  |  |  | |  |
| 1. Poorest | 3.5 | 42.6 | | 24.2 | 28.8 | 4.46 | |  |
| 2 | 17.5 | 15.8 | | 16.5 | 25.6 | 20.5 | |  |
| 3 | 33.3 | 12.9 | | 13.4 | 14.4 | 32.1 | |  |
| 4 | 30.7 | 8.9 | | 16.1 | 23.2 | 17.0 | |  |
| 5- Least poor | 14.9 | 19.8 | | 26.9 | 8.0 | 25.9 | | 118.8, 16 (<0.001) |
