# Supplementary material for: Measuring Coverage in MNCH: Accuracy of Measuring Diagnosis and Treatment of Childhood Malaria from Household Surveys in Zambia
Source: PLoS Med. 2013 May 7;10(5):e1001417. doi: 10.1371/journal.pmed.1001417 (PMC3646207; doi:10.1371/journal.pmed.1001417)
Supplement: Table S2 — Accuracy of caregiver recall of key questions of diagnosis and treatment of malaria for children with reported fever in the past 2 wk, by follow-up and social-demographic characteristics, Western Province, Zambia, 2012. (DOC) [file pmed.1001417.s002.doc]

**Table S2: Accuracy of caregiver recall of key questions of diagnosis and treatment of malaria for children with reported fever in the past 2 weeks, by follow-up and social-demographic characteristics, Western Province Zambia 2012**

| **Caregiver recall** | **Sensitivity**  **(%)** | **95% confidence interval** | **Specificity**  **(%)** | **95% confidence interval** | **Accuracy**  **(%)** | **95% confidence interval** | **Sample size** |
| --- | --- | --- | --- | --- | --- | --- | --- |
| **Recall finger/heel stick** |  |  |  |  |  |  |  |
| Facility |  |  |  |  |  |  |  |
| Kahare | 39.2 | 29.5 – 48.9** | 100 | - | 48.3 | 39.1 – 57.4** | 114 |
| Luampa | 18.6 | 9.5 – 27.7** | 90.9 | 73.9 – 100 | 28.4 | 18.6 – 38.2** | 81 |
| Mulamba | 14.3 | 0 – 40.2** | 89.9 | 84.3 – 94.9 | 86.3 | 80.7 – 91.9** | 146 |
| Mwanambuyu | 94.2 | 89.7 – 98.7** | 81.0 | 64.2 – 97.8 | 91.9 | 87.1 – 96.7** | 124 |
| Nkeyema | 85.6 | 79.1 – 92.1** | 100 | - | 85.7 | 79.2 – 92.2** | 112 |
| Child age in years |  |  |  |  |  |  |  |
| 0 | 58.6 | 48.3 – 69.0 | 88.2 | 79.4 – 97.1 | 69.6 | 61.9 – 77.2 | 138 |
| 1 | 57.9 | 48.8 – 67.0 | 87.5 | 78.1 – 96.9 | 66.7 | 59.4 – 73.9 | 162 |
| 2 | 67.9 | 57.8 – 78.1 | 94.7 | 87.6 – 99.4 | 76.5 | 68.9 – 84.1 | 119 |
| 3 | 68.8 | 57.4 – 80.1 | 92.3 | 85.1 – 99.1 | 75.6 | 66.7 – 84.4 | 90 |
| 4 | 66.7 | 52.4 – 80.9 | 88.5 | 76.2 – 97.6 | 75.0 | 64.7 – 85.3 | 68 |
| Child sex |  |  |  |  |  |  |  |
| Male | 69.1 | 62.6 – 75.6* | 88.9 | 82.4 – 95.4 | 75.4 | 70.3 – 80.4 | 284 |
| Female | 56.7 | 49.7 – 63.7* | 90.9 | 85.3 – 96.8 | 68.3 | 62.9 – 73.6 | 293 |
| Respondent at home |  |  |  |  |  |  |  |
| Mother | 63.6 | 58.5 – 68.5 | 91.5 | 87.2 – 95.7 | 72.1 | 68.3 – 75.9 | 538 |
| Caregiver other than mother | 42.9 | 16.9 – 68.8 | 80.0 | 64.3 – 95.7 | 66.7 | 51.9 – 81.5 | 39 |
| Days to follow-up |  |  |  |  |  |  |  |
| 0-6 | 59.9 | 54.1 – 65.7 | 92.6 | 87.7 – 97.5 | 69.1 | 64.5 – 73.7* | 382 |
| 7-14 | 70.2 | 61.8 – 78.6 | 86.4 | 79.0 – 93.9 | 76.9 | 71.0 – 82.8* | 195 |
| SES |  |  |  |  |  |  |  |
| 1. Poorest | 58.2 | 46.4 – 70.2 | 85.4 | 75.4 – 95.4 | 69.6 | 61.2 – 77.8 | 115 |
| 2 | 67.1 | 56.7 – 77.5 | 94.6 | 87.8 – 100 | 75.9 | 68.1 – 83.6 | 116 |
| 3 | 65.3 | 55.7 – 74.8 | 96.2 | 88.8 – 100 | 71.9 | 63.9 – 79.9 | 121 |
| 4 | 64.6 | 54.0 – 75.1 | 85.7 | 74.1 – 97.3 | 71.1 | 62.7 – 79.4 | 114 |
| 5- Least poor | 57.4 | 45.6 – 69.1 | 90.7 | 82.0 – 99.4 | 70.3 | 61.8 – 78.8 | 111 |
| Mother age |  |  |  |  |  |  |  |
| 18-24 | 64.2 | 56.9 – 71.6 | 87.1 | 79.3 – 95.0 | 71.1 | 65.3 – 76.9 | 235 |
| 25-34 | 63.5 | 55.8 – 71.3 | 94.1 | 89.0 – 98.0 | 74.6 | 69.0 – 80.2 | 232 |
| 35-44 | 58.6 | 47.0 – 70.1 | 91.7 | 80.6 – 100 | 67.0 | 57.5 – 76.5 | 94 |
| ≥45 | 60.0 | 17.1 – 100 | 72.7 | .46 – 99.1 | 68.8 | 46.0 – 89.0 | 16 |
| Education |  |  |  |  |  |  |  |
| None | 50.0 | 28.1 – 71.9 | 83.3 | 66.1 – 100 | 65.8 | 50.7 – 80.9 | 38 |
| At least some primary | 64.9 | 58.9 – 70.9 | 90.9 | 85.3 – 96.6 | 72.5 | 67.8 – 77.3 | 338 |
| Secondary or higher | 61.2 | 52.8 – 69.7 | 90.3 | 83.4 – 97.1 | 71.6 | 65.4 – 77.9 | 201 |
| **Total** | **62.9** | **58.1 – 67.7** | **90.0** | **85.7 – 94.2** | **71.8** | **68.1 – 75.4** | **577** |
|  |  |  |  |  |  |  |  |
| **Recall positive malaria test result (of those tested at clinic)** |  |  |  |  |  |  |  |
| Facility |  |  |  |  |  |  |  |
| Kahare | 25.5 | 13.1 – 38.0** | 98.0 | 94.1 – 100** | 62.9 | 53.3 – 72.5** | 97 |
| Luampa | 18.0 | 5.9 – 30.0** | 90.3 | 79.9 – 100** | 50.0 | 38.3 – 61.7** | 70 |
| Mulamba | 33.3 | 0 – 86.7** | 100 | -** | 71.4 | 38.0 – 100** | 7 |
| Mwanambuyu | 96.2 | 91.1 – 100** | 96.0 | 90.6 – 100** | 96.1 | 92.4 – 99.9** | 103 |
| Nkeyema | 83.3 | 75.4 – 91.3** | 66.7 | 48.9 – 84.5** | 79.3 | 71.7 – 86.8** | 111 |
| Child age in years |  |  |  |  |  |  |  |
| 0 | 53.1 | 35.8 – 70.4 | 94.6 | 88.5 – 98.9 | 79.3 | 70.8 – 87.8 | 87 |
| 1 | 53.5 | 40.6 – 66.3 | 91.1 | 83.6 – 98.6 | 71.9 | 63.7 – 80.2 | 114 |
| 2 | 61.4 | 48.8 – 74.0 | 87.5 | 74.3 – 97.3 | 69.1 | 59.1 – 79.2 | 81 |
| 3 | 75.0 | 62.8 – 87.3 | 81.3 | 62.1 – 96.0 | 76.6 | 66.2 – 86.9 | 64 |
| 4 | 71.0 | 55.0 – 87.0 | 90.9 | 73.9 – 99.8 | 76.2 | 63.3 – 89.1 | 42 |
| Child sex |  |  |  |  |  |  |  |
| Male | 66.9 | 58.6 – 75.3 | 91.8 | 85.5 – 98.1 | 76.3 | 70.3 – 82.3 | 194 |
| Female | 57.1 | 47.7 – 66.6 | 89.9 | 83.6 – 96.2 | 72.2 | 65.9 – 78.5 | 194 |
| Respondent at home |  |  |  |  |  |  |  |
| Mother | 64.2 | 57.8 – 70.6* | 90.6 | 86.0 – 95.1 | 75.4 | 71.0 – 79.8 | 374 |
| Caregiver other than mother | 27.3 | 0.1 – 53.6* | 100 | - | 42.9 | 16.9 – 68.8 | 14 |
| Days to follow-up |  |  |  |  |  |  |  |
| 0-6 | 57.4 | 49.6 – 65.2* | 88.2 | 82.5 – 94.0 | 70.8 | 65.4 – 76.2* | 274 |
| 7-14 | 73.2 | 62.9 – 83.5* | 97.7 | 93.2 – 100 | 82.5 | 75.5 – 89.4* | 114 |
| SES |  |  |  |  |  |  |  |
| 1. Poorest | 59.5 | 43.6 – 75.3 | 90.0 | 79.3 – 100 | 73.1 | 62.5 – 83.8 | 67 |
| 2 | 71.1 | 57.9 – 84.4 | 94.1 | 86.2 – 100 | 81.0 | 72.4 – 89.7 | 79 |
| 3 | 65.5 | 52.9 – 78.0 | 90.0 | 80.7 – 99.3 | 75.8 | 67.2 – 84.4 | 95 |
| 4 | 60.4 | 46.6 – 74.3 | 90.3 | 79.9 – 100 | 72.2 | 62.3 – 82.0 | 79 |
| 5- Least poor | 53.7 | 38.4 – 68.9 | 88.9 | 77.0 – 100 | 67.7 | 56.5 – 78.8 | 68 |
| Mother age |  |  |  |  |  |  |  |
| 18-24 | 67.8 | 58.0 – 77.6 | 92.3 | 86.4 – 98.2 | 79.4 | 73.2 – 85.6 | 165 |
| 25-34 | 61.1 | 51.3 – 70.9 | 84.9 | 75.3 – 94.5 | 69.6 | 62.2 – 77.0 | 148 |
| 35-44 | 55.0 | 39.6 – 70.4 | 96.7 | 90.1 – 100 | 72.9 | 62.4 – 83.3 | 70 |
| ≥45 |  |  |  |  |  |  | 5 |
| Education |  |  |  |  |  |  |  |
| None | 66.7 | 35.9 – 97.5 | 90.9 | 73.9 – 100 | 80.0 | 62.5 – 97.5 | 20 |
| At least some primary | 59.2 | 51.1 – 67.2 | 91.8 | 86.3 – 97.2 | 72.4 | 66.7 – 78.1 | 239 |
| Secondary or higher | 68.0 | 57.4 – 78.6 | 88.9 | 80.5 – 97.3 | 76.7 | 69.5 – 84.0 | 129 |
| **Total** | **62.4** | **56.1 – 68.7** | **90.7** | **86.3 – 95.2** | **74.2** | **69.9 – 78.6** | **388** |
|  |  |  |  |  |  |  |  |
| **Recall that malaria diagnosis was made*** |  |  |  |  |  |  |  |
| Facility |  |  |  |  |  |  |  |
| Kahare | 87.5 | 78.1 – 96.9** | 95.5 | 90.4 – 100** | 92.1 | 87.2 – 97.1** | 114 |
| Luampa | 75.0 | 60.9 – 89.1** | 48.9 | 34.3 – 63.5** | 60.5 | 49.9 – 71.1** | 81 |
| Mulamba | 58.8 | 49.7 – 67.8** | 53.1 | 35.8 – 70.4** | 57.5 | 49.5 – 65.6** | 146 |
| Mwanambuyu | 90.6 | 83.5 – 97.8** | 88.3 | 80.2 – 96.5** | 89.5 | 84.1 – 94.9** | 124 |
| Nkeyema | 85.5 | 78.0 – 93.1** | 72.4 | 56.2 – 88.7** | 82.1 | 75.1 – 89.2** | 112 |
| Child age in years |  |  |  |  |  |  |  |
| 0 | 71.2 | 59.6 – 82.7 | 77.2 | 68.0 – 86.5 | 74.6 | 67.4 – 81.9 | 138 |
| 1 | 81.3 | 72.7 – 89.8 | 70.7 | 60.9 – 80.6 | 75.9 | 69.3 – 82.5 | 162 |
| 2 | 72.9 | 63.5 – 82.4 | 82.4 | 69.5 – 95.2 | 75.6 | 67.9 – 83.3 | 119 |
| 3 | 79.4 | 69.8 – 89.0 | 86.4 | 72.0 – 97.1 | 81.1 | 73.0 – 89.2 | 90 |
| 4 | 79.3 | 68.3 – 90.2 | 66.7 | 42.8 – 90.5 | 76.5 | 66.4 – 86.6 | 68 |
| Child sex |  |  |  |  |  |  |  |
| Male | 74.7 | 68.3 – 81.1 | 75.5 | 67.3 – 86.7 | 75.0 | 70.0 – 80.0 | 284 |
| Female | 79.0 | 72.9 – 85.2 | 76.2 | 68.8 – 83.6 | 77.8 | 73.1 – 82.6 | 293 |
| Respondent at home |  |  |  |  |  |  |  |
| Mother | 76.4 | 71.3 – 81.1 | 75.9 | 70.3 – 81.5 | 76.2 | 72.6 – 79.8 | 538 |
| Caregiver other than mother | 80.7 | 66.7 – 94.6 | 75.0 | 44.5 – 100 | 79.5 | 66.8 – 92.2 | 39 |
| Days to follow-up |  |  |  |  |  |  |  |
| 0-6 | 75.0 | 69.1 – 80.9 | 78.1 | 72.0 – 83.9 | 76.4 | 72.2 – 80.7 | 382 |
| 7-14 | 79.4 | 72.3 – 86.1 | 68.5 | 56.1 – 80.9 | 76.4 | 70.5 – 82.4 | 195 |
| SES |  |  |  |  |  |  |  |
| 1. Poorest | 78.8 | 68.9 – 88.7 | 63.3 | 49.8 – 76.8* | 72.2 | 64.0 – 80.4 | 115 |
| 2 | 78.7 | 68.4 – 89.0 | 70.9 | 58.9 – 82.9* | 75.0 | 67.1 – 82.9 | 116 |
| 3 | 81.8 | 73.2 – 90.4 | 90.9 | 82.4 – 99.4* | 85.1 | 78.8 – 91.5 | 121 |
| 4 | 75.4 | 65.2 – 85.6 | 82.2 | 71.1 – 93.4* | 78.1 | 70.5 – 85.7 | 114 |
| 5- Least poor | 69.4 | 58.8 – 80.1 | 74.4 | 60.7 – 88.1* | 71.2 | 62.7 – 79.6 | 111 |
| Mother age |  |  |  |  |  |  |  |
| 18-24 | 77.8 | 70.5 – 85.0 | 76.2 | 68.2 – 84.2 | 77.0 | 71.6 – 82.4 | 235 |
| 25-34 | 77.5 | 70.8 – 84.2 | 74.1 | 64.5 – 83.6 | 76.3 | 70.8 – 81.8 | 232 |
| 35-44 | 74.6 | 63.0 – 86.1 | 79.5 | 66.8 – 92.2 | 76.6 | 68.0 – 85.2 | 94 |
| ≥45 | 69.2 | 44.1 – 94.3 | 66.7 | 13.3 – 100 | 68.8 | 46.0 – 91.5 | 16 |
| Education |  |  |  |  |  |  |  |
| None | 81.8 | 65.7 – 97.9 | 50.0 | 25.5 – 74.5* | 68.4 | 53.6 – 83.2 | 38 |
| At least some primary | 75.4 | 69.4 – 81.3 | 80.7 | 74.1 – 87.4* | 77.5 | 73.1 – 82.0 | 338 |
| Secondary or higher | 78.3 | 71.0 – 85.7 | 72.8 | 63.2 – 82.5* | 76.1 | 70.2 – 82.0 | 201 |
| **Total** | **76.8** | **72.4 – 81.3** | **75.9** | **70.4 – 81.4*** | **76.4** | **73.0 – 79.9** | **577** |
|  |  |  |  |  |  |  |  |
| **ACT given** |  |  |  |  |  |  |  |
| Facility |  |  |  |  |  |  |  |
| Kahare | 35.6 | 21.6 – 49.5** | 98.6 | 95.7 – 100** | 73.7 | 65.6 – 81.8** | 114 |
| Luampa | 43.2 | 27.3 – 59.2** | 93.2 | 85.7 – 100** | 70.4 | 60.4 – 80.3** | 81 |
| Mulamba | 97.5 | 94.7 – 100** | 66.7 | 48.9 – 84.5** | 91.8 | 87.3 – 96.2** | 146 |
| Mwanambuyu | 93.4 | 87.2 – 99.7** | 93.7 | 87.6 – 99.7** | 93.6 | 89.2 – 97.9** | 124 |
| Nkeyema | 90.0 | 83.4 – 96.6** | 90.6 | 80.5 – 100** | 90.2 | 84.7 – 95.7** | 112 |
| Child age in years |  |  |  |  |  |  |  |
| 0 | 82.1 | 72.1 – 92.2 | 95.1 | 90.5 – 99.8 | 89.9 | 84.8 – 94.9 | 138 |
| 1 | 78.8 | 70.1 – 87.5 | 92.2 | 86.2 – 98.2 | 85.2 | 79.7 – 90.7 | 162 |
| 2 | 83.5 | 75.6 – 91.4 | 91.2 | 81.6 – 98.1 | 85.7 | 79.4 – 92.0 | 119 |
| 3 | 81.0 | 71.3 – 90.7 | 85.2 | 71.8 – 98.6 | 82.2 | 74.3 – 90.1 | 90 |
| 4 | 79.3 | 98.3 – 90.2 | 80.0 | 59.8 – 95.7 | 79.4 | 69.8 – 89.0 | 68 |
| Child sex |  |  |  |  |  |  |  |
| Male | 81.7 | 76.0 – 87.3 | 90.4 | 84.7 – 96.1 | 84.9 | 80.7 – 89.0 | 284 |
| Female | 80.3 | 74.1 – 86.4 | 92.4 | 87.8 – 96.9 | 85.7 | 81.7 – 89.7 | 293 |
| Respondent at home |  |  |  |  |  |  |  |
| Mother | 81.0 | 76.6 – 85.3 | 92.1 | 88.6 – 95.6 | 86.7 | 82.7 – 88.7 | 538 |
| Caregiver other than mother | 81.3 | 67.7 – 94.8 | 71.4 | 38.1 – 100 | 79.5 | 66.8 – 92.2 | 39 |
| Days to follow-up |  |  |  |  |  |  |  |
| 0-6 | 76.7 | 70.9 – 82.6* | 93.9 | 90.4 – 97.4* | 84.8 | 81.2 – 88.4 | 382 |
| 7-14 | 87.1 | 81.6 – 92.7* | 83.6 | 73.9 – 93.4* | 86.2 | 81.3 – 91.0 | 195 |
| SES |  |  |  |  |  |  |  |
| 1. Poorest | 78.8 | 68.9 – 88.7 | 93.9 | 87.2 – 100 | 85.2 | 78.7 – 91.7 | 115 |
| 2 | 84.1 | 75.1 – 93.2 | 90.6 | 82.7 – 98.4 | 87.1 | 81.0 – 93.2 | 116 |
| 3 | 79.4 | 69.8 – 89.0 | 90.6 | 82.7 – 96.9 | 84.3 | 77.8 – 90.8 | 121 |
| 4 | 72.9 | 62.4 – 83.3 | 90.9 | 82.4 – 99.4 | 79.8 | 72.5 – 86.8 | 114 |
| 5- Least poor | 89.3 | 82.4 – 96.3 | 91.7 | 82.6 – 100 | 90.1 | 84.5 – 95.7 | 111 |
| Mother age |  |  |  |  |  |  |  |
| 18-24 | 82.0 | 75.2 – 88.8 | 91.2 | 85.9 – 96.4 | 86.4 | 82.0 – 90.8 | 235 |
| 25-34 | 80.3 | 73.9 – 86.6 | 91.3 | 85.1 – 97.4 | 84.1 | 79.3 – 88.8 | 232 |
| 35-44 | 80.4 | 70.0 – 90.8 | 97.4 | 92.3 – 100 | 87.2 | 80.5 – 94.0 | 94 |
| ≥45 | 83.3 | 62.3 – 100 | 50.0 | 6.8 – 93.2 | 75.0 | 53.8 – 96.2 | 16 |
| Education |  |  |  |  |  |  |  |
| None | 81.0 | 64.1 – 97.8 | 82.4 | 64.2 – 100 | 81.6 | 69.3 – 93.9 | 38 |
| At least some primary | 78.7 | 73.1 – 84.4 | 94.1 | 90.2 – 97.4 | 84.9 | 81.1 – 88.7 | 338 |
| Secondary or higher | 84.9 | 78.4 – 91.3 | 89.0 | 82.3 – 95.8 | 86.6 | 81.9 – 91.3 | 201 |
| **Total** | **81.0** | **76.8 – 85.2** | **91.5** | **87.9 – 95.1** | **85.3** | **82.4 – 88.2** | **577** |

*p-value < 0.05; *** p-value < 0.001
