# Supplementary material for: Measuring Coverage in MNCH: Accuracy of Measuring Diagnosis and Treatment of Childhood Malaria from Household Surveys in Zambia
Source: PLoS Med. 2013 May 7;10(5):e1001417. doi: 10.1371/journal.pmed.1001417 (PMC3646207; doi:10.1371/journal.pmed.1001417)
Supplement: Table S3 — Bivariate statistical test for differences of accuracy of caregiver recall of key questions of diagnosis and treatment of malaria for children with reported fever in the past 2 wk, by follow-up and social-demographic characteristics, Western Province, Zambia, 2012. (DOC) [file pmed.1001417.s003.doc]

**Table S3: Bivariate statistical test for differences of accuracy of caregiver recall of key questions of diagnosis and treatment of malaria for children with reported fever in the past 2 weeks, by follow-up and social-demographic characteristics, Western Province Zambia 2012**

| **Caregiver recall** | **Sensitivity** | | **Specificity** | | **Accuracy** | |
| --- | --- | --- | --- | --- | --- | --- |
|  | Chi-square  test statistic | p-value | Chi-square  test statistic | p-value | Chi-square  test statistic | p-value |
| **Recall finger/heel stick** | n = 388 | | n = 189 | | n = 577 | |
| Facility (df = 4) | 157.06 | <0.001* | 3.90 | 0.3687* | 157.14 | <0.001 |
| Child age (df =4 ) | 3.97 | 0.4103 | 1.67 | 0.8171* | 4.70 | 0.3199 |
| Child sex (df =1) | 6.36 | 0.0117 | 0.22 | 0.2093* | 3.58 | 0.0585 |
| Mother or caregiver (df = 1) | 2.50 | 0.1574* | 3.15 | 0.1430* | 0.53 | 0.4652 |
| Days to follow-up (df = 1) | 3.67 | 0.0552 | 1.95 | 0.1625 | 3.89 | 0.0486 |
| SES (df = 4) | 2.44 | 0.6550 | 3.80 | 0.4336 | 1.39 | 0.8464 |
| Mother age (df = 3) | 0.73 | 0.8413* | 5.86 | 0.1067* | 2.07 | 0.5354* |
| Education (df = 2) | 1.97 | 0.3736 | 0.98 | 0.5863* | 0.76 | 0.6848 |
| **Recall positive malaria test result (of those tested at clinic)** | n = 226 | | n = 162 | | N = 388 | |
| Facility (df = 4) | 102.68 | <0.001* | 21.34 | <0.001* | 59.52 | <0.001* |
| Child age (df =4 ) | 7.40 | 0.1151* | 2.97 | 0.4534* | 2.85 | 0.5825 |
| Child sex | 2.30 | 0.1293 | 0.17 | 0.7886* | 0.86 | 0.3531 |
| Mother or caregiver (df = 1) | 6.08 | 0.0220* | 2.32 | 0.1711* | 4.13 | 0.0622 |
| Days to follow-up (df = 1) | 5.19 | 0.0227 | 3.35 | 0.1200* | 5.71 | 0.0168 |
| SES (df = 4) | 3.23 | 0.5208 | 0.63 | 0.9606 | 3.78 | 0.4364 |
| Mother age (df = 3) | 2.37 | 0.4859* | 3.73 | 0.2705* | 4.56 | 0.1586* |
| Education (df = 2) | 1.71 | 0.4178* | 0.34 | 0.8283* | 1.20 | 0.6351* |
| **Recall that malaria diagnosis was made*** | n = 345 | | n = 232 | | n = 577 | |
| Facility (df = 4) | 34.38 | <0.001* | 46.03 | <0.001* | 69.72 | <0.001* |
| Child age (df =4 ) | 3.08 | 0.5442 | 4.06 | 0.4127 | 1.41 | 0.8432 |
| Child sex | 0.90 | 0.3417 | 0.02 | 0.8986 | 0.63 | 0.4256 |
| Mother or caregiver (df = 1) | 0.28 | 0.8234* | 0.01 | 0.9538* | 0.22 | 0.8451* |
| Days to follow-up (df = 1) | 0.92 | 0.3375 | 2.07 | 0.1499* | 0.01 | 0.9937 |
| SES (df = 4) | 3.62 | 0.4592 | 11.47 | 0.0176* | 8.24 | 0.0832 |
| Mother age (df = 3) | 0.68 | 0.8190* | 0.56 | 0.8463* | 0.57 | 0.8790* |
| Education (df = 2) | 0.70 | 0.7467* | 8.00 | 0.0203* | 1.58 | 0.4313 |
| **ACT given** | n = 342 | | n = 235 | | n = 577 | |
| Facility (df = 4) | 125.97 | <0.001* | 26.36 | <0.001* | 40.34 | <0.001* |
| Child age (df =4 ) | 0.77 | 0.9426 | 5.37 | 0.1948* | 4.85 | 0.3028 |
| Child sex | 0.11 | 0.7383 | 0.29 | 0.6422* | 0.07 | 0.7847 |
| Mother or caregiver (df = 1) | 0.01 | 0.9691* | 3.73 | 0.1116* | 1.11 | 0.3458* |
| Days to follow-up (df = 1) | 5.82 | 0.0158 | 5.69 | 0.0257* | 0.18 | 0.6682 |
| SES (df = 4) | 7.12 | 0.1228 | 0.50 | 0.9763* | 5.13 | 0.2738 |
| Mother age (df = 3) | 0.19 | 0.9793* | 10.6 | 0.0507* | 2.14 | 0.5076* |
| Education (df = 2) | 1.85 | 0.3808* | 3.67 | 0.1352* | 0.72 | 0.6483* |

Df = degrees of freedom; *Fishers exact test used due to small cell counts
