# Supplementary material for: Measuring Coverage in MNCH: Accuracy of Measuring Diagnosis and Treatment of Childhood Malaria from Household Surveys in Zambia
Source: PLoS Med. 2013 May 7;10(5):e1001417. doi: 10.1371/journal.pmed.1001417 (PMC3646207; doi:10.1371/journal.pmed.1001417)
Supplement: Table S4 — Random effects logistic regression models of sensitivity, specificity, and accuracy of caregiver recall of key questions of diagnosis and treatment of malaria for children with reported fever in the past 2 wk: associations with follow-up and social-demographic characteristics, Western Province, Zambia, 2012. (DOC) [file pmed.1001417.s004.doc]

**Table S4: Random effects logistic regression models of sensitivity, specificity and accuracy of caregiver recall of key questions of diagnosis and treatment of malaria for children with reported fever in the past 2 weeks: associations with follow-up and social-demographic characteristics, Western Province Zambia 2012**

|  |  | **Sensitivity** |  |  | **Specificity** |  |  | **Accuracy** |  |
| --- | --- | --- | --- | --- | --- | --- | --- | --- | --- |
| **Caregiver recall** | AOR | (95% CI) | p-value | AOR | (95% CI) | p-value | AOR | (95% CI) | p-value |
| **Recall finger/heel stick** |  | n = 388 |  |  | n = 189 |  |  | n = 577 |  |
| Child age in years |  |  |  |  |  |  |  |  |  |
| 0 | 0.92 | (0.80 – 1.06) | 0.2402 | 0.99 | (0.85 – 1.15) | 0.8706 | 0.95 | (0.85 – 1.06) | 0.3772 |
| 1 | 0.97 | (0.84 – 1.11) | 0.6163 | 0.98 | (0.85 – 1.14) | 0.8298 | 0.98 | (0.88 – 1.10) | 0.7268 |
| 2 | 1.03 | (0.89 – 1.18) | 0.6676 | 1.07 | (0.92 – 1.25) | 0.6172 | 1.04 | (0.93 – 1.17) | 0.4615 |
| 3 | 0.98 | (0.85 – 1.14) | 0.8747 | 1.04 | (0.88 – 1.24) | 0.6172 | 1.00 | (0.88 – 1.13) | 0.9544 |
| 4 | 1 |  |  | 1 |  |  | 1 |  |  |
| Male vs. female (reference) | 1.10 | (1.02 – 1.19) | 0.0111 | 1.00 | (0.92 – 1.08) | 0.9631 | 1.05 | (0.99 – 1.12) | 0.1229 |
| Mother vs. caregiver (reference) | 1.04 | (0.91 – 1.20) | 0.5428 | 1.10 | (0.94 – 1.27) | 0.2332 | 1.07 | (0.93 – 1.23) | 0.3228 |
| Days to follow-up: 0-6 vs. 7-14 days (reference) | 1.01 | (0.92 – 1.10) | 0.8877 | 1.07 | (0.98 – 1.18) | 0.1455 | 1.05 | (0.98 – 1.13) | 0.1589 |
| SES |  |  |  |  |  |  |  |  |  |
| 1. Poorest | 1.04 | (0.91 – 1.20) | 0.5428 | 0.93 | (0.82 – 1.07) | 0.3131 | 1.02 | (0.92 – 1.14) | 0.6589 |
| 2 | 1.02 | (0.89 – 1.16) | 0.8027 | 1.03 | (0.90 – 1.18) | 0.6652 | 1.03 | (0.93 – 1.14) | 0.6067 |
| 3 | 1.06 | (0.94 – 1.20) | 0.3248 | 1.01 | (0.87 – 1.18) | 0.8709 | 1.02 | (0.92 – 1.13) | 0.6917 |
| 4 | 1.06 | (0.93 – 1.20) | 0.3828 | 0.92 | (0.80 – 1.06) | 0.2335 | 1.01 | (0.91 – 1.11) | 0.9155 |
| 5- Least poor | 1 |  |  | 1 |  |  | 1 |  |  |
| Mother age |  |  |  |  |  |  |  |  |  |
| 18-24 | 1.08 | (0.77 – 1.51) | 0.6403 | 1.10 | (0.88 – 1.37) | 0.4121 | 1.11 | (0.90 – 1.36) | 0.3330 |
| 25-34 | 1.03 | 0.74 – 1.44) | 0.8510 | 1.19 | (0.97 – 1.47) | 0.0991 | 1.11 | (0.90 – 1.36) | 0.3221 |
| 35-44 | 0.99 | (0.70 – 1.39) | 0.9428 | 1.17 | (0.94 – 1.47) | 0.1605 | 1.04 | (0.85 – 1.30) | 0.6599 |
| ≥45 | 1 |  |  | 1 |  |  | 1 |  |  |
| Education |  |  |  |  |  |  |  |  |  |
| None | 0.89 | (0.74 – 1.07) | 0.2045 | 0.91 | (0.76 – 1.07) | 0.2536 | 0.91 | (0.79 – 1.05) | 0.2084 |
| At least some primary | 0.97 | (0.89 – 1.07) | 0.5999 | 0.98 | (0.89 – 1.08) | 0.6872 | 0.99 | (0.92 – 1.06) | 0.7885 |
| Secondary or higher | 1 |  |  | 1 |  |  | 1 |  |  |
|  |  |  |  |  |  |  |  |  |  |
| **Recall positive malaria test result (of those tested at clinic)** |  | n = 226 |  |  | n = 162 |  |  | n = 388 |  |
| Child age in years |  |  |  |  |  |  |  |  |  |
| 0 | 0.80 | (0.66 – 0.96) | 0.0173 | 1.04 | (0.86 – 1.25) | 0.7072 | 0.97 | (0.83 – 1.13) | 0.7123 |
| 1 | 0.84 | (0.72 – 1.00) | 0.0463 | 0.95 | (0.79 – 1.15) | 0.6173 | 0.94 | (0.81 – 1.09) | 0.4373 |
| 2 | 0.89 | (0.76 – 1.05) | 0.1611 | 0.93 | (0.75 – 1.14) | 0.4653 | 0.92 | (0.79 – 1.08) | 0.3100 |
| 3 | 0.94 | (0.79 – 1.11) | 0.4588 | 0.88 | (0.70 – 1.09) | 0.2445 | 0.95 | (0.81 – 1.12) | 0.5668 |
| 4 | 1 |  |  | 1 |  |  | 1 |  |  |
| Male vs. female (reference) | 1.04 | (0.94 – 1.15) | 0.4112 | 0.97 | (0.89 – 1.06) | 0.4851 | 1.01 | (0.93 – 1.10) | 0.7341 |
| Mother vs. caregiver (reference) | 1.06 | (0.83 – 1.36) | 0.6017 | 0.94 | (0.68 – 1.31) | 0.7188 | 1.25 | (0.99 – 1.58) | 0.0559 |
| Days to follow-up: 0-6 vs. 7-14 days (reference) | 0.98 | (0.88 – 1.10) | 0.7451 | 0.88 | (0.79 – 0.97) | 0.0126 | 0.91 | (0.82 – 0.99) | 0.0386 |
| SES |  |  |  |  |  |  |  |  |  |
| 1. Poorest | 1.03 | (0.87 – 1.23) | 0.7005 | 0.95 | (0.81 – 1.13) | 0.5710 | 1.03 | (0.88 – 1.19) | 0.7373 |
| 2 | 1.03 | (0.88 – 1.23) | 0.7126 | 0.98 | (0.83 – 1.15) | 0.7643 | 1.07 | (0.92 – 1.23) | 0.3598 |
| 3 | 1.05 | (0.90 – 1.23) | 0.5086 | 0.97 | (0.84 – 1.11) | 0.6285 | 1.05 | (0.92 – 1.20) | 0.4603 |
| 4 | 1.11 | (0.95 – 1.30) | 0.1967 | 0.98 | (0.85 – 1.15) | 0.8370 | 0.99 | (0.87 – 1.14) | 0.9099 |
| 5- Least poor | 1 |  |  | 1 |  |  | 1 |  |  |
| Mother age |  |  |  |  |  |  |  |  |  |
| 18-24 | 1.11 | (0.76 – 1.63) | 0.5817 | 0.88 | (0.50 – 1.54) | 0.6497 | 1.25 | (0.86 – 1.81) | 0.2404 |
| 25-34 | 1.07 | (0.73 – 1.56) | 0.7350 | 0.81 | (0.46 – 1.42) | 0.4599 | 1.13 | (0.78 – 1.63) | 0.5105 |
| 35-44 | 1.04 | (0.71 – 1.53) | 0.8432 | 0.93 | (0.52 – 1.63) | 0.7879 | 1.21 | (0.83 – 1.76) | 0.3163 |
| ≥45 | 1 |  |  | 1 |  |  | 1 |  |  |
| Education |  |  |  |  |  |  |  |  |  |
| None | 0.93 | (0.71 – 1.22) | 0.6165 | 0.95 | (0.78 – 1.16) | 0.6039 | 0.96 | (0.79 – 1.18) | 0.7182 |
| At least some primary | 0.89 | (0.80 – 1.00) | 0.0514 | 0.96 | (0.87 – 1.07) | 0.4963 | 0.89 | (0.81 – 0.98) | 0.0216 |
| Secondary or higher | 1 |  |  | 1 |  |  | 1 |  |  |
|  |  |  |  |  |  |  |  |  |  |
| **Recall that malaria diagnosis was made*** | n = 345 |  |  | n = 232 |  |  | n = 577 |  |  |
| Child age in years |  |  |  |  |  |  |  |  |  |
| 0 | 0.94 | (0.81 – 1.09) | 0.4066 | 1.05 | (0.84 – 1.31) | 0.6562 | 0.97 | (0.86 – 1.10) | 0.6576 |
| 1 | 1.04 | (0.90 – 1.19) | 0.6184 | 1.01 | (0.81 – 1.26) | 0.9285 | 1.00 | (0.89 – 1.12) | 0.9531 |
| 2 | 0.92 | (0.80 – 1.06) | 0.2424 | 1.14 | (0.89 – 1.46) | 0.2937 | 0.98 | (0.87 – 1.11) | 0.7974 |
| 3 | 0.97 | (0.84 – 1.12) | 0.6748 | 1.21 | (0.93 – 1.58) | 0.1605 | 1.04 | (0.91 – 1.18) | 0.5857 |
| 4 | 1 |  |  | 1 |  |  | 1 |  |  |
| Male vs. female (reference) | 0.95 | (0.87 – 1.04) | 0.2569 | 0.98 | (0.88 – 1.09) | 0.6981 | 0.97 | (0.91 – 1.04) | 0.3748 |
| Mother vs. caregiver (reference) | 0.82 | (0.69 – 0.96) | 0.0140 | 1.06 | (0.77 – 1.46) | 0.7259 | 0.88 | (0.76 – 1.02) | 0.0930 |
| Days to follow-up: 0-6 vs. 7-14 days (reference) | 0.89 | (0.81 – 0.98) | 0.0126 | 1.07 | (0.94 – 1.22) | 0.3182 | 0.96 | (0.89 – 1.03) | 0.2834 |
| Laboratory diagnosis vs. clinical (reference) | 1.41 | (1.28 – 1.56) | <0.001 | 1.08 | (0.96 – 1.22) | 0.2015 | 1.01 | (0.90 – 1.13) | 0.9013 |
| SES |  |  |  |  |  |  |  |  |  |
| 1. Poorest | 1.11 | (0.97 – 1.28) | 0.1300 | 0.86 | (0.72 – 1.03) | 0.1077 | 1.05 | (0.94 – 1.17) | 0.4195 |
| 2 | 1.10 | (0.95 – 0.21) | 0.2076 | 0.85 | (0.71 – 1.02) | 0.0759 | 1.00 | (0.90 – 1.12) | 0.9496 |
| 3 | 1.08 | (0.94 – 1.24) | 0.2331 | 1.01 | (0.84 – 1.21) | 0.9340 | 1.07 | (0.96 – 1.20) | 0.2067 |
| 4 | 1.04 | (0.91 – 1.19) | 0.5914 | 0.91 | (0.76 – 1.09) | 0.3080 | 1.00 | (0.89 – 1.11) | 0.9660 |
| 5- Least poor | 1 |  |  | 1 |  |  | 1 |  |  |
| Mother age () |  |  |  |  |  |  |  |  |  |
| 18-24 | 1.06 | (0.83 – 1.35) | 0.6424 | 1.11 | (0.67 – 1.84) | 0.6910 | 1.04 | (0.84 – 1.30) | 0.7183 |
| 25-34 | 1.07 | (0.84 – 1.35) | 0.5927 | 1.08 | (0.65 – 1.78) | 0.7848 | 1.04 | (0.84 – 1.29) | 0.7043 |
| 35-44 | 1.01 | (0.78 – 1.29) | 0.9643 | 1.08 | (0.65 – 1.78) | 0.7722 | 1.02 | (0.81 – 1.28) | 0.8526 |
| ≥45 | 1 |  |  | 1 |  |  | 1 |  |  |
| Education |  |  |  |  |  |  |  |  |  |
| None | 1.05 | (0.87 – 1.28) | 0.6111 | 0.88 | (0.70 – 1.11) | 0.2719 | 0.95 | (0.82 – 1.11) | 0.5228 |
| At least some primary | 0.95 | (0.86 – 1.05) | 0.3139 | 1.06 | (0.94 – 1.20) | 0.3350 | 0.98 | (0.91 – 1.06) | 0.6479 |
| Secondary or higher | 1 |  |  | 1 |  |  | 1 |  |  |
|  |  |  |  |  |  |  |  |  |  |
| **ACT given** |  | n = 342 |  |  | n = 235 |  |  | n = 577 |  |
| Child age in years |  |  |  |  |  |  |  |  |  |
| 0 | 1.04 | (0.92 – 1.18) | 0.5115 | 1.12 | (0.97 – 1.31) | 0.1257 | 1.09 | (0.99 – 1.21) | 0.0904 |
| 1 | 1.02 | (0.91 – 1.14) | 0.7354 | 1.05 | (0.90 – 1.22) | 0.5274 | 1.06 | (0.96 – 1.17) | 0.2284 |
| 2 | 1.07 | (0.96 – 1.20) | 0.2130 | 1.06 | (0.89 – 1.25) | 0.5254 | 1.07 | (0.96 – 1.18) | 0.2101 |
| 3 | 1.03 | (0.91 – 1.16) | 0.6266 | 1.04 | (0.87 – 1.23) | 0.6745 | 1.03 | (0.92 – 1.14) | 0.6418 |
| 4 | 1 |  |  | 1 |  |  | 1 |  |  |
| Male vs. female (reference) | 1.02 | (0.95 – 1.09) | 0.6265 | 0.98 | (0.91 – 1.05) | 0.5749 | 0.98 | (0.93 – 1.04) | 0.5223 |
| Mother vs. caregiver (reference) | 1.01 | (0.89 – 1.15) | 0.8827 | 1.10 | (0.87 – 1.37) | 0.4314 | 1.06 | (0.93 – 1.20) | 0.3940 |
| Days to follow-up: 0-6 vs. 7-14 days (reference) | 1.02 | (0.95 – 1.10) | 0.5917 | 1.04 | (0.95 – 1.13) | 0.3674 | 1.02 | (0.96 – 1.08) | 0.5681 |
| Laboratory diagnosis vs. clinical (reference) | 1.06 | (0.97 – 1.15) | 0.1749 | 1.01 | (0.92 – 1.10) | 0.8877 | 1.17 | (1.08 – 1.26) | 0.002 |
| SES |  |  |  |  |  |  |  |  |  |
| 1. Poorest | 0.92 | (0.82 – 1.02) | 0.1209 | 1.04 | (0.92 – 1.18) | 0.5023 | 0.95 | (0.86 – 1.05) | 0.2910 |
| 2 | 0.88 | (0.79 – 0.99) | 0.0318 | 0.97 | (0.86 – 1.10) | 0.6343 | 0.95 | (0.87 – 1.04) | 0.2952 |
| 3 | 0.91 | (0.82 – 1.02) | 0.1058 | 0.92 | (0.81 – 1.03) | 0.1551 | 0.95 | (0.87 – 1.04) | 0.3034 |
| 4 | 0.92 | (0.82 – 1.01) | 0.0904 | 0.96 | (0.85 – 1.09) | 0.5384 | 0.89 | (0.82 – 0.98) | 0.0188 |
| 5- Least poor | 1 |  |  | 1 |  |  | 1 |  |  |
| Mother age () |  |  |  |  |  |  |  |  |  |
| 18-24 | 1.01 | (0.82 – 1.23) | 0.9562 | 1.24 | (0.92 – 1.68) | 0.1502 | 1.13 | (0.94 – 1.36) | 0.1961 |
| 25-34 | 1.02 | (0.83 – 1.24) | 0.8754 | 1.28 | (0.95 – 1.72) | 0.1031 | 1.10 | (0.91 – 1.32) | 0.3219 |
| 35-44 | 1.04 | (0.85 – 1.28) | 0.6881 | 1.36 | (1.01 – 1.83) | 0.0445 | 1.16 | (0.96 – 1.40) | 0.1225 |
| ≥45 | 1 |  |  | 1 |  |  | 1 |  |  |
| Education |  |  |  |  |  |  |  |  |  |
| None | 0.96 | (0.82 – 1.13) | 0.6316 | 0.92 | (0.79 – 1.06) | 0.2513 | 0.96 | (0.84 – 1.09) | 0.5142 |
| At least some primary | 0.98 | (0.90 – 1.05) | 0.5239 | 1.04 | (0.96 – 1.13) | 0.3162 | 0.98 | (0.92 – 1.05) | 0.6682 |
| Secondary or higher | 1 |  |  | 1 |  |  | 1 |  |  |

AOR: Adjusted odds ratio

CI: Confidence interval

Facility included as a random effect in all models
